# Adjunctive transcranial direct current stimulation for cognitive improvement in schizophrenia: insights from a systematic review and exploratory meta-analysis

**DOI:** 10.3389/fpsyt.2025.1617068

**Published:** 2025-07-11

**Authors:** Gaolei Yao, Yuan Huang, Junjiao Ping, Yixuan Han, Liming Lu, Xinxia Liu, Huipeng Lu

**Affiliations:** ^1^ Clinical Research and Big Data Laboratory, South China Research Center for Acupuncture and Moxibustion, Medical College of Acu-Moxi and Rehabilitation, Guangzhou University of Chinese Medicine, Guangzhou, China; ^2^ Department of Psychiatry, The Third People’s Hospital of Zhongshan, Zhongshan, China; ^3^ Department of Acupuncture, Shaoguan Hospital of Traditional Chinese Medicine, Shaoguan, China; ^4^ European Languages and Cultures, Beijing Foreign Studies University, Beijing, China

**Keywords:** transcranial direct current stimulation, cognitive function, schizophrenia, systematic review, meta-analysis

## Abstract

**Introduction:**

To objectively and accurately evaluate the clinical efficacy of transcranial direct current stimulation (tDCS) for schizophrenia, we conducted this systematic review and exploratory meta-analysis (PROSPERO: CRD420251002632).

**Methods:**

Firstly, randomized controlled trials (RCTs) related to the adjunctive use of tDCS for schizophrenia were searched on PubMed, Embase and Web of Science databases according to search strategies developed in advance. Secondly, the eligible articles were screened according to the inclusion and exclusion criteria. Finally, the data were extracted and a complete meta-analysis was performed. Meanwhile, we also completed publication bias analysis and sensitivity analysis. Statistical analysis was conducted using RevMan software (version 5.4).

**Results:**

We ultimately included 11 eligible RCTs. The results of the exploratory meta-analysis indicated that the adjunctive use of tDCS seems to be able to enhance the cognitive function of patients (the Paced Auditory Serial Addition Task: standardized mean deviation (*SMD*) = 3.57, 95% confidence interval (*CI*) (2.60, 4.54), *P* < 0.00001). Subgroup analyses showed that the adjunctive use of tDCS at 15 sessions (Clinical Global Impression-Schizophrenia scale: *SMD*= -0.56, 95% *CI* (-0.87, -0.25), *P*=0.0004) or twice daily (Clinical Global Impression-Schizophrenia scale: *SMD*= -0.85, 95% *CI* (-1.67, -0.03), *P*=0.04) also decreased the disease severity.

**Discussion:**

This exploratory meta-analysis revealed that tDCS in the treatment of schizophrenia appears capable of improving cognitive function and effectively reducing the severity of the disease. What’s more, tDCS has great development potential in schizophrenia, and it may be written into the guidelines for non-drug treatment of schizophrenia in the future. As a matter of fact, this exploratory study points out the direction for future scientific research and clinical practice.

**Systematic review registration:**

https://www.crd.york.ac.uk/PROSPERO/, identifier CRD420251002632.

## 1 Introduction

Schizophrenia is a common severe mental illness. As of 2022, approximately 24 million people worldwide suffer from schizophrenia (1). The large number of people affected by schizophrenia is the first major difficulty that schizophrenia poses to families and society. Patients with acute schizophrenia are prone to excessive behavior, which brings harm to their families and society. Correspondingly, people with chronic schizophrenia have a higher rate of disability later in life (2). Antipsychotic therapy is the mainline of treatment for schizophrenia; However, approximately 1/3 of patients do not respond to treatment (3). This is the second major difficulty that schizophrenia brings to society. In fact, even patients who have a clear response to existing drug treatments and experience symptom relief still have a significant risk of relapse when faced with stressors (4). The high recurrence rate is the third major challenge in completely overcoming schizophrenia. Moreover, the pathological mechanism of schizophrenia is quite complex, involving metabolic disorders and abnormal protein function (5). Cognitive dysfunction is a core symptom of schizophrenia, and this irreversible damage to neurological function poses a significant challenge to the recovery of patients (6). The above challenges have brought great pressure on patients with schizophrenia, their families and even society. Therefore, there is an urgent need to optimize existing treatment options and find more efficient treatment measures in the field of psychiatry.

Despite the fact that the majority of antipsychotics have an impact on the mental symptoms experienced by individuals diagnosed with schizophrenia, they are ineffective in the recovery of cognitive function (7). This is particularly evident in their inability to enhance working memory deficits in patients. The cognitive decline is multifaceted, encompassing symptoms such as memory loss, impaired learning ability, and inattention (8). A significant proportion of patients diagnosed with schizophrenia, specifically 85%, exhibit cognitive impairment (9).

Transcranial electrical stimulation has long been used in psychiatric diseases (10), and studies have confirmed that the adjunctive use of transcranial alternating current stimulation can alleviate both total and negative neuropsychiatric symptoms in patients with schizophrenia (11). Similarly, transcranial direct current stimulation (tDCS) is also a common non-invasive treatment and is currently widely used in Parkinson’s disease (12, 13), stroke (14, 15), depression (16–18), attention-deficit/hyperactivity disorder (19), insomnia (20) and impairment of cognitive function (21, 22). In recent years, tDCS is also considered to have the characteristics of improving delusion and social function of schizophrenia patients (23, 24). In addition, some scholars have found that tDCS can relieve the negative symptoms of schizophrenia patients and improve working memory (25). However, not all tDCS have obtained satisfactory scientific research results. Previous studies have also revealed negative results of tDCS in the treatment of schizophrenia (26, 27). Therefore, in the face of confused results, it is difficult for clinicians to determine the efficacy of tDCS in schizophrenia, let alone make direct clinical decisions. Although the efficacy of tDCS in schizophrenia has been explored in recent years by meta-analyses (26, 28, 29), especially demonstrating its potential to improve working memory deficits in patients (29), however, the studies included in these meta-analyses confounded schizophrenia, schizoaffective disorder, delusional disorder, affective disorder, and borderline personality disorder, and therefore, we believe that methodological flaws impair the reliability of the conclusions of these meta-analyses.

In order to fill this research gap, we hypothesized that tDCS may improve cognitive function in schizophrenia, and conducted this systematic review and exploratory meta-analysis to evaluate the clinical efficacy of tDCS in schizophrenia, providing a reference for clinicians to develop treatment plans and policymakers to formulate schizophrenia treatment policies.

## 2 Method

Under the guidance of the Preferred Reporting Items for Systematic Reviews and Meta-Analyses (PRISMA), we have completed the process of literature retrieval and screening, literature quality evaluation, data extraction and analysis. This research plan was registered in PROSPERO (https://www.crd.york.ac.uk/PROSPERO/home) in advance (CRD420251002632).

### 2.1 Search strategy

We searched the PubMed, Embase and Web of Science (WOS) databases for tDCS studies related to schizophrenia, with the literature spanning from the time of library construction to March 2025, and developed search strategies through a combination of subject and free terms (see **Supplementary Material**). The main subject terms include: “schizophrenias”, “dementia praecox”, “schizophrenic disorders”, “disorder, schizophrenic”, “disorders, schizophrenic”, “schizophrenic disorder”, “anodal stimulation transcranial direct current stimulation”, “cathodal stimulation transcranial direct current stimulation”, “transcranial alternating current stimulation”, “repetitive transcranial electrical stimulation”, “transcranial electrical stimulation”. Two researchers independently conducted literature searches, and any disagreements were resolved by a senior literature research expert. Relevant literature was retrieved using a predefined search strategy, and the searched documents were then imported into NoteExpress (version 4.1.0) software.

### 2.2 Inclusion and exclusion criteria

#### 2.2.1 Inclusion criteria

1. Research type: Randomized controlled trial studies.
2. Participants: Patients with schizophrenia diagnosed by the Diagnostic and Statistical Manual of Mental Disorders (DSM) or International Classification of Diseases (ICD)-10.
3. Interventions: tDCS alone or tDCS + basic therapy.
4. Control: basic therapy.
5. Outcomes: the Positive and Negative Syndrome Scale (PANSS), the Scale for the Assessment of Negative Symptoms (SANS), the Auditory Hallucinations Rating Scale (AHRS), Calgary Depression Scale for Schizophrenia (CDSS), Clinical Global Impression-Schizophrenia scale (CGI), the Paced Auditory Serial Addition Task (PASAT).
6. Language: English.

#### 2.2.2 Exclusion criteria

1. Directly repeating literature or publishing literature with duplicated data.
2. Literature involving other diseases or literature not related to tDCS research.
3. Literature in the form of reviews and letters.
4. Animal researches.
5. Review, Mendelian randomization study, protocol, case report.
6. The data are incomplete or the data cannot be extracted.
7. Documents unable to obtain full text.
8. self-control study before and after.

### 2.3 Data extraction

After the final inclusion of the literature was determined according to the screening criteria, two researchers began to independently extract relevant information of the included literature. Firstly, record the extracted author names, publication years, titles, diagnostic criteria, interventions, sessions and frequency of interventions, and outcome measures in an Excel spreadsheet. In case of discrepancies, a third researcher will be invited to discuss and reach a consensus. In this meta-analysis, the PASAT, a cognitive function evaluation indicator, was utilized as the primary outcome indicator, with the remaining indicators serving as secondary outcome indicators.

### 2.4 Quality assessment

Systematic review was carried out using the methodology and quality evaluation standards of the Cochrane handbook, and the quality evaluation of 7 items for each included literature was carried out (30): random sequence generation, allocation concealment, blinding, outcome assessment bias, incomplete reporting, selective reporting, other biases, each item is rated on 3 levels: low risk, high risk, and unclear. If an item in the literature is evaluated as low risk or high risk, it indicates that it is impossible to change the result seriously or the result can be questioned, and if it is unclear, the scientific research credibility of the result will be seriously weakened. Furthermore, the Jadad scale was utilized to evaluate the quality of the included articles. The total score is determined by the quality of the submission, with 1–2 points allocated for low quality and 3–5 points for high quality. The Jadad scale is a tool designed to assess the methodological quality of randomized controlled trials in clinical research. It focuses on four key aspects: randomization, blinding, withdrawal and dropouts. Finally, we also used grade evaluation to assess each outcome.

### 2.5 Statistical analyses

Statistical analyses were performed applying RevMan software (Cochrane Review Manager Version 5.4, Oxford, UK) provided by the Cochrane Collaboration Network, and measurements were expressed as standardized mean deviation (*SMD*) and 95% confidence intervals (95% *CI*). To test the heterogeneity of the included research literature, when the test results were *P* ≥ 0.1 or *I* ^2^ < 50, a fixed effect model was used for meta-analysis; Otherwise, a random effects model was used for the exploratory meta-analysis and attempts were made to analyze sources of heterogeneity (30). Funnel plot was drawn by software, and the included literature was analyzed for publication bias according to whether the funnel plot was symmetrically distributed or not. The leave one out method was used to sequentially exclude each included study to evaluate the robustness of the combined effect of the remaining studies through sensitivity analysis (30). Previous studies have found that treatment sessions and intervention frequency can affect the clinical efficacy of tDCS (26), so, this study attempts to conduct subgroup analysis based on treatment sessions and intervention frequency.

## 3 Results

### 3.1 Literature search and study selection

**Figure 1:**
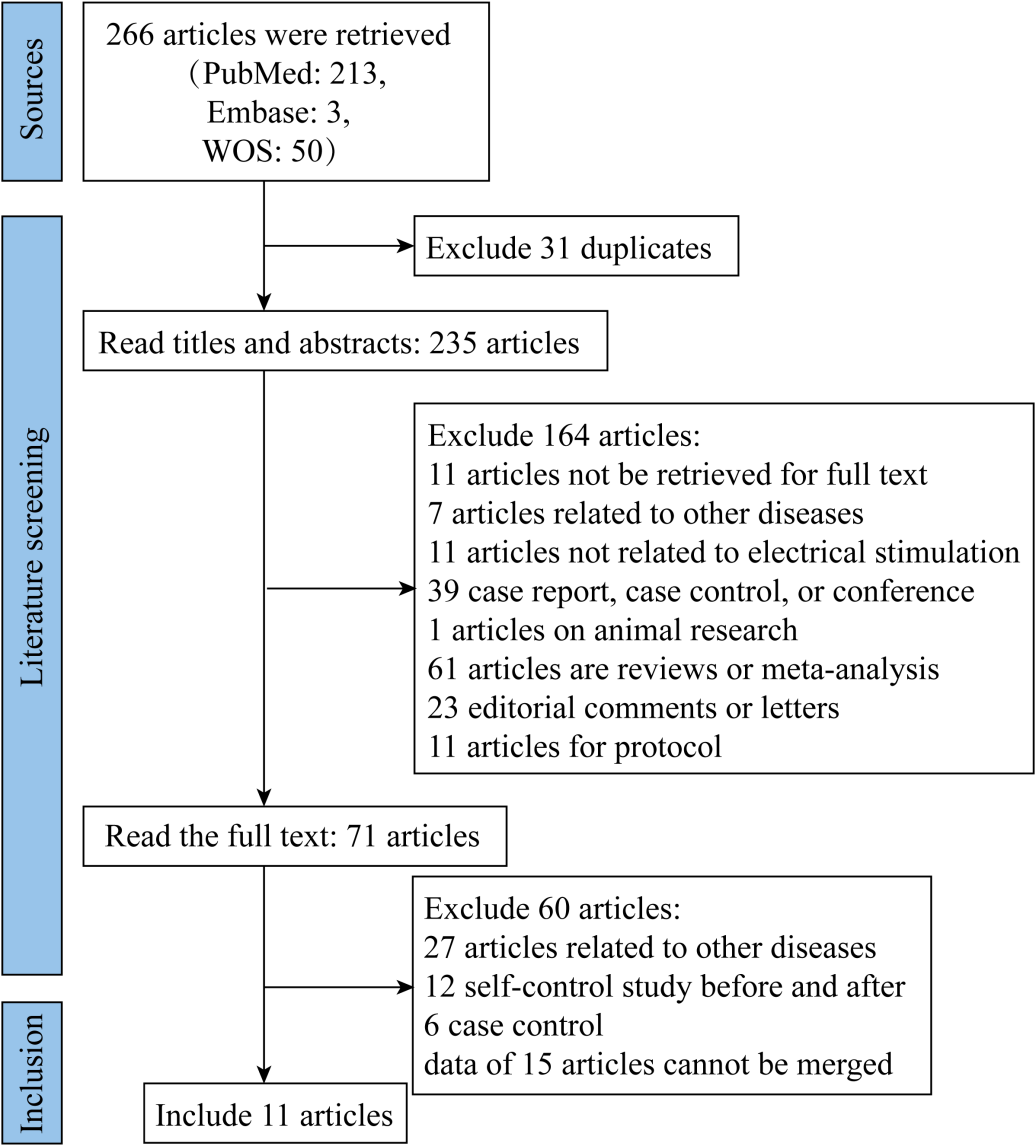
Flowchart of Literature Retrieval and Screening. WOS, Web of Science.

Based on the pre-specified inclusion and exclusion criteria, we retrieved relevant literature from PubMed, Embase and WOS databases, respectively. After removing duplicate documents and reading titles and abstracts, 71 documents were read in full, and 11 articles were finally included in this meta-analysis (25, 31–40) (**Figure 1**).

### 3.2 Study characteristics

The 11 articles included in this exploratory study involved a total of 468 individuals, with 240 in the active tDCS group and 228 in the sham tDCS group. The sample size for a single article varied from 16 to 100. The years of publication included in the literature were 2016 to 2024, and the intervention was 10 sessions or 15 sessions. What’s more, patients included in 4 articles included left handedness and right handedness (25, 31, 33, 39), 6 articles only included right handedness (32, 34–36, 38, 40), however, the remaining 1 article did not mention handedness (37). In addition, 1 article clearly stated that patients with paranoid schizophrenia or disorganized schizophrenia were recruited (39). The basic characteristics of the included literature are shown in **Table 1**.

**Table 1:** Basic characteristics of included literature.

Author	Year	Diagnostic criteria	NO. intervention^a^	Intervention	NO. control^a^	Control	Sessions and frequency	Outcome
Mondino, Marine (33)	2016	DSM-IV-TR	11	active tDCS+medications	12	sham tDCS+medications	10 sessions, twice daily	AHRS
Palm, Ulrich (39)	2016	DSM-IV	10	active tDCS+medications	10	sham tDCS+medications	10 sessions, once daily	PANSS, SANS
Jeon, Dong-Wook (31)	2018	DSM-V	28	active tDCS+medications	28	sham tDCS+medications	10 sessions, once daily	PANSS, SANS, CDSS, CGI
Bose, Anushree (38)	2018	DSM-IV	12	active tDCS+medications	13	sham tDCS+medications	10 sessions twice daily	AHRS
Smith, Robert C (35)	2020	DSM-V or DSM-IV	24	active tDCS+medications	25	sham tDCS+medications	10 sessions, once daily	PANSS, SANS, PASAT
Valiengo, Leandro Da Costa Lane (37)	2020	DSM-IV	50	active tDCS+medications	50	sham tDCS+medications	10 sessions twice daily	PANSS, SANS, AHRS, CDSS
Dharani, Ramamoorthy (32)	2021	ICD-10	8	active tDCS+medications	8	sham tDCS+medications	10 sessions twice daily	SANS, CGI
Lisoni, Jacopo (25)	2022	DSM-V	25	active tDCS+medications	25	sham tDCS+medications	15 sessions, once daily	PANSS, SANS, CGI
Liu, Yong (34)	2022	DSM-V	16	active tDCS+medications	11	sham tDCS+medications	10 sessions, once daily	PASAT
Zhou, Yue (36)	2023	DSM-IV	21	active tDCS+medications	17	sham tDCS+medications	15 sessions, once daily	PANSS, SANS
Lyu, Xiaoli (40)	2024	DSM-IV	35	active tDCS+medications	29	sham tDCS+medications	15 sessions, once daily	PANSS, SANS

^a^Data were extracted based on random assignment. DSM-IV, the Diagnostic and Statistical Manual of Mental Disorder, fourth edition; DSM-V, the Diagnostic and Statistical Manual of Mental Disorder, fifth edition; DSM-IV-TR, the Diagnostic and Statistical Manual of Mental Disorders, fourth edition, text revision; ICD-10, International Classification of Diseases, 10th revision; tDCS, transcranial direct current stimulation; AHRS, the Auditory Hallucinations Rating Scale; PANSS, the Positive and Negative Syndrome Scale; SANS, the Scale for the Assessment of Negative Symptoms; CDSS, Calgary Depression Scale for Schizophrenia; CGI, Clinical Global Impression-Schizophrenia scale; PASAT, the Paced Auditory Serial Addition Task.

### 3.3 Quality assessment

**Figure 2:**
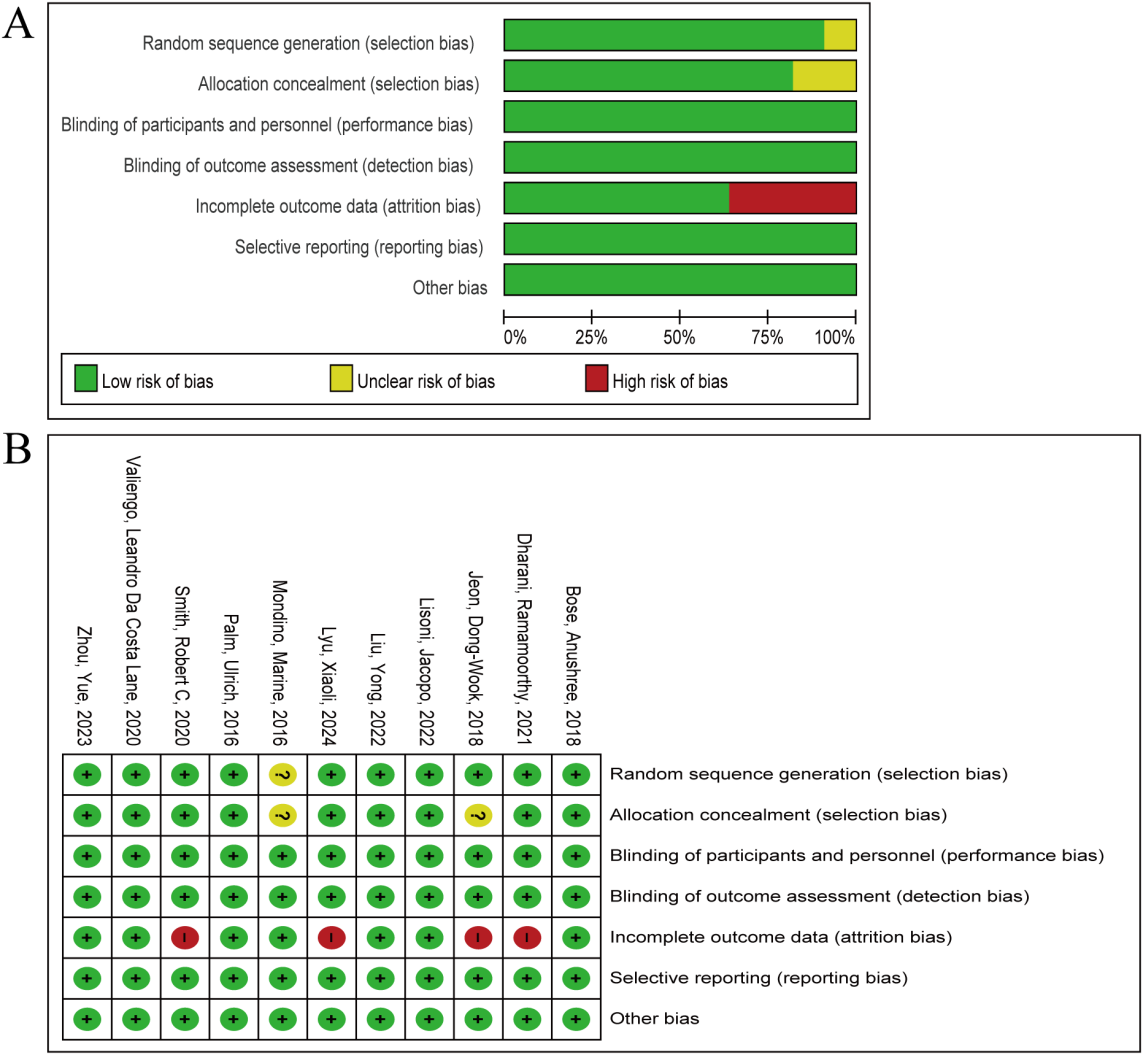
Summary of risk of bias in the meta-analysis. **(A)** the percentage of ratings for 11 articles across seven items. **(B)** The score distribution of each article on seven items. Green, yellow and red represent low risk of bias, unclear risk of bias and high risk of bias respectively.

The 11 literature included in this exploratory study showed overall high quality, with low risk of bias occupying the main position in all seven dimensions (**Figure 2A**). Ten articles pointed out clear random grouping methods, such as computer-generated random number list (25, 34–40), block randomization (31), enveloped randomization (32), however, the remaining one paper only proposed randomization, but did not report the specific randomization method (33). The implementation of the nine studies conformed to the principle of allocation concealment (25, 32, 34–40), the remaining two studies did not report assignment concealment (31, 33). All studies implemented appropriate blinding procedures. Four studies generated dropout cases (31, 35, 38, 40), which could lead to attrition bias (**Figure 2B**). Six articles reported adverse events (25, 35–37, 39, 40). Moreover, 7 articles have completed follow-up (31, 32, 34, 35, 37, 39, 40). Utilizing the Jadad scale score, two articles were categorized as low quality, while the remaining articles were designated as high quality (**Table 2**). Grade reviews found that Pasat and CDSS had Moderate and High levels of evidence, respectively, and other outcomes were low (**Supplementary Table 1**).

**Table 2:** The results of Jadad scale score.

Author	Year	Randomization	Double blinding	Withdrawals and dropouts	Total scores
Mondino, Marine (33)	2016	1	2	0	3
Palm, Ulrich (39)	2016	2	2	1	5
Jeon, Dong-Wook (31)	2018	2	2	0	4
Bose, Anushree (38)	2018	2	2	1	5
Smith, Robert C (35)	2020	2	0	0	2
Valiengo, Leandro Da Costa Lane (37)	2020	2	2	1	5
Dharani, Ramamoorthy (32)	2021	2	2	1	5
Lisoni, Jacopo (25)	2022	2	2	1	5
Liu, Yong (34)	2022	2	0	0	2
Zhou, Yue (36)	2023	2	2	0	4
Lyu, Xiaoli (40)	2024	2	2	1	5

Two articles were classified as low quality, while the remaining articles were designated as high quality.

### 3.4 Primary outcome

#### 3.4.1 PASAT

**Figure 3:**
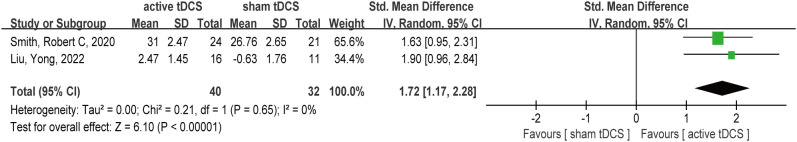
Forest plot of tDCS for schizophrenia measured by PASAT. At the PASAT levels, active tDCS showed a significant intervention advantage. tDCS, transcranial direct current stimulation; CI, confidence interval; PASAT, the Paced Auditory Serial Addition Task.

Two articles reported post-intervention PASAT results (10 sessions, once daily) (31, 32), involving 72 subjects, including 40 and 32 in active tDCS and sham tDCS, respectively. The heterogeneity test results showed no significant heterogeneity (*P*= 0.65, I^2^ = 0%), however, given the small sample size, so the random effect model was selected. The results showed that the pooled effect size *SMD*= 1.72, 95% CI (1.17, 2.28), *P*<0.00001 (**Figure 3**). The exploratory analysis results showed that at the PASAT level, active tDCS seemed to be able to show intervention advantages.

### 3.5 Secondary outcomes

#### 3.5.1 PANSS

**Figure 4:**
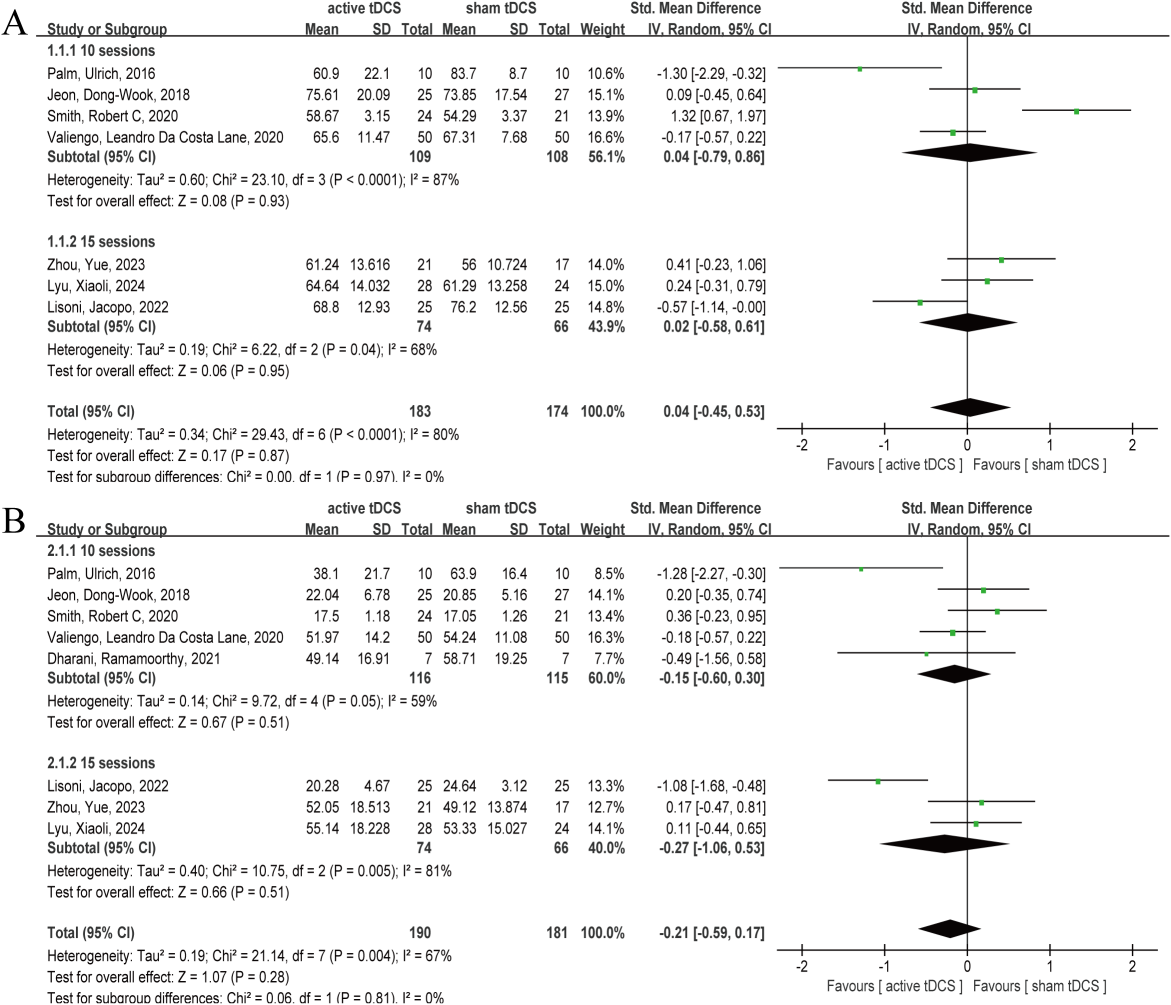
Forest plot of tDCS for schizophrenia measured by PANSS and SANS. At the PANSS and SANS levels, active tDCS did not show a significant intervention advantage. **(A)** PANSS, **(B)** SANS. tDCS, transcranial direct current stimulation; CI, confidence interval; PANSS, the Positive and Negative Syndrome Scale; SANS, the Scale for the Assessment of Negative Symptoms.

Seven articles reported PANSS results after intervention (25, 31, 35–37, 39, 40), involving 357 participants, including 183 in active tDCS and 174 in sham tDCS, respectively. The heterogeneity test results showed significant heterogeneity (*P*=0.0001, *I* ^2^ = 80%), so the random effect model was selected. The analysis results showed that the combined effect size *SMD*=0.04, 95% *CI* (-0.45, 0.53), *P*=0.87 (**Figure 4A**). The analysis results showed that, at the PANSS level, active tDCS did not show a significant intervention advantage and there was significant heterogeneity (*P*=0.0001, *I* ^2^ = 80%). Therefore, we attempted to explore the sources of heterogeneity by the sessions of treatment and the frequency of intervention. Active tDCS did not show a significant intervention advantage in the 10 sessions and 15 sessions subgroups (*P*=0.93, *P*=0.95), nor did active tDCS at once daily and twice daily (*P*=0.81, *P*=0.39).

#### 3.5.2 SANS

Eight articles reported post-intervention SANS outcomes (25, 31, 32, 35–37, 39, 40), involving 371 subjects, with 190 and 181 in active tDCS and sham tDCS, respectively. Heterogeneity test results showed that there was obvious heterogeneity (*P*=0.004, *I* ^2^ = 67%), so the random effect model was selected. The analysis results showed that the pooled effect size *SMD*= -0.21, 95% *CI* (-0.59, 0.17), *P*=0.28 (**Figure 4B**). The results of the analysis showed that, at the SANS level, active tDCS did not show a clear intervention advantage, and there was obvious heterogeneity (*P*=0.004, *I* ^2^ = 67%). Therefore, we attempted to explore the sources of heterogeneity with sessions and frequency of intervention. In the subgroups of 10 and 15 treatment sessions, active tDCS did not demonstrate a significant intervention advantage (*P*=0.51, *P*=0.51), nor did active tDCS at both once daily and twice daily frequencies (*P*=0.46, *P*=0.27).

#### 3.5.3 AHRS

**Figure 5:**
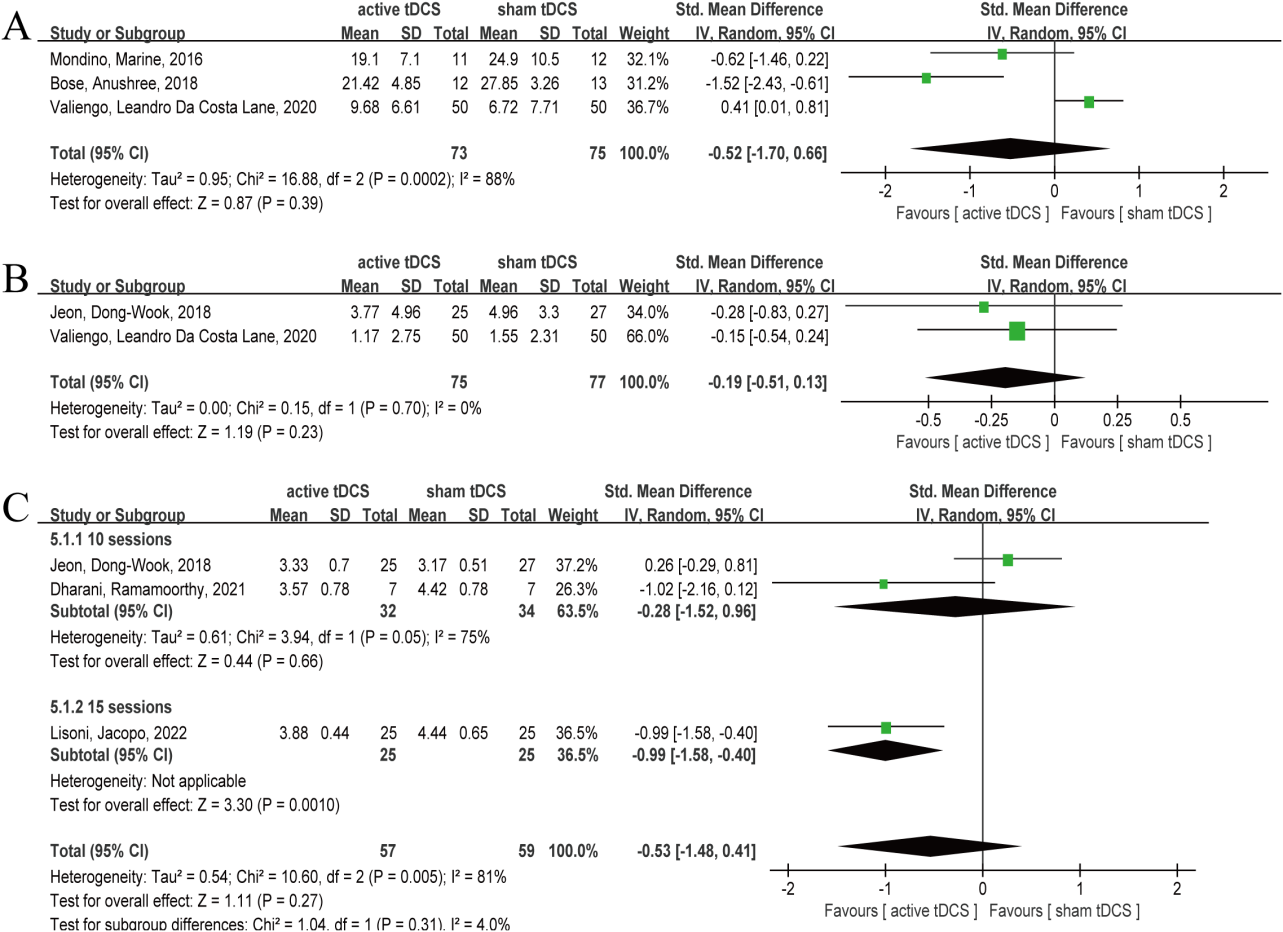
Forest plot of tDCS for schizophrenia measured by AHRS, CDSS and CGI. At the AHRS, CDSS and CGI levels, active tDCS did not show a significant intervention advantage. **(A)** AHRS, **(B)** CDSS, **(C)** CGI. tDCS, transcranial direct current stimulation; CI, confidence interval; AHRS, the Auditory Hallucinations Rating Scale; CDSS, Calgary Depression Scale for Schizophrenia; CGI, Clinical Global Impression-Schizophrenia scale.

Three articles reported the results of AHRS after 10 sessions intervention (twice daily) (33, 37, 38), involving 148 subjects, including 73 in active tDCS and 75 in sham tDCS, respectively. Heterogeneity test showed significant heterogeneity (*P*=0.0002, *I* ^2^ = 88%), therefore, random effect model was selected. The analysis results showed that the combined effect *SMD*= -0.52, 95% *CI* (-1.70, 0.66), *P*=0.39 (**Figure 5A**). The results of the analysis showed that at the AHRS level, active tDCS did not show a significant intervention advantage, and there was significant heterogeneity (*P*=0.0002, *I* ^2^ = 88%).

#### 3.5.4 CDSS

Two articles reported the CDSS results after 10 sessions intervention (31, 37), involving 152 subjects, including 75 and 77 in active tDCS and sham tDCS respectively. The heterogeneity test results showed that there was no significant heterogeneity (*P*=0.70, *I* ^2^ = 0%), however, given the small sample size, so the random effect model was selected. The results showed that the pooled effect size was *SMD*= -0.19, 95% *CI* (-0.51, 0.13), *P*=0.23 (**Figure 5B**). The analysis results showed that active tDCS did not show significant intervention advantages at the CDSS level.

#### 3.5.5 CGI

Three articles reported CGI outcomes after intervention (25, 31, 32), involving 116 subjects, with 57 and 59 subjects in active tDCS and sham tDCS, respectively. The heterogeneity test results showed that there was significant heterogeneity (*P*=0.005, *I* ^2^ = 81%), therefore, a random effect model was selected. The analysis results showed combined effect size *SMD*= -0.53, 95% *CI* (-1.48, 0.41), *P*=0.27 (**Figure 5C**). The analysis results indicated that at the CGI level, active tDCS did not show a significant intervention advantage, and there was considerable heterogeneity (*P*=0.005, *I* ^2^ = 81%). Therefore, we attempted to explore the sources of heterogeneity using treatment sessions and intervention frequency. In the subgroup of 15 sessions, active tDCS showed a significant intervention advantage (*P*=0.001), whereas in the subgroup of 10 sessions, active tDCS and sham tDCS did not show a statistical difference (*P*=0.66). On the frequency of twice daily, active tDCS showed obvious intervention advantages (*P*=0.04), while on the frequency of once daily, active tDCS failed to show obvious intervention advantages (*P*=0.57).

### 3.6 Publication bias

**Figure 6:**
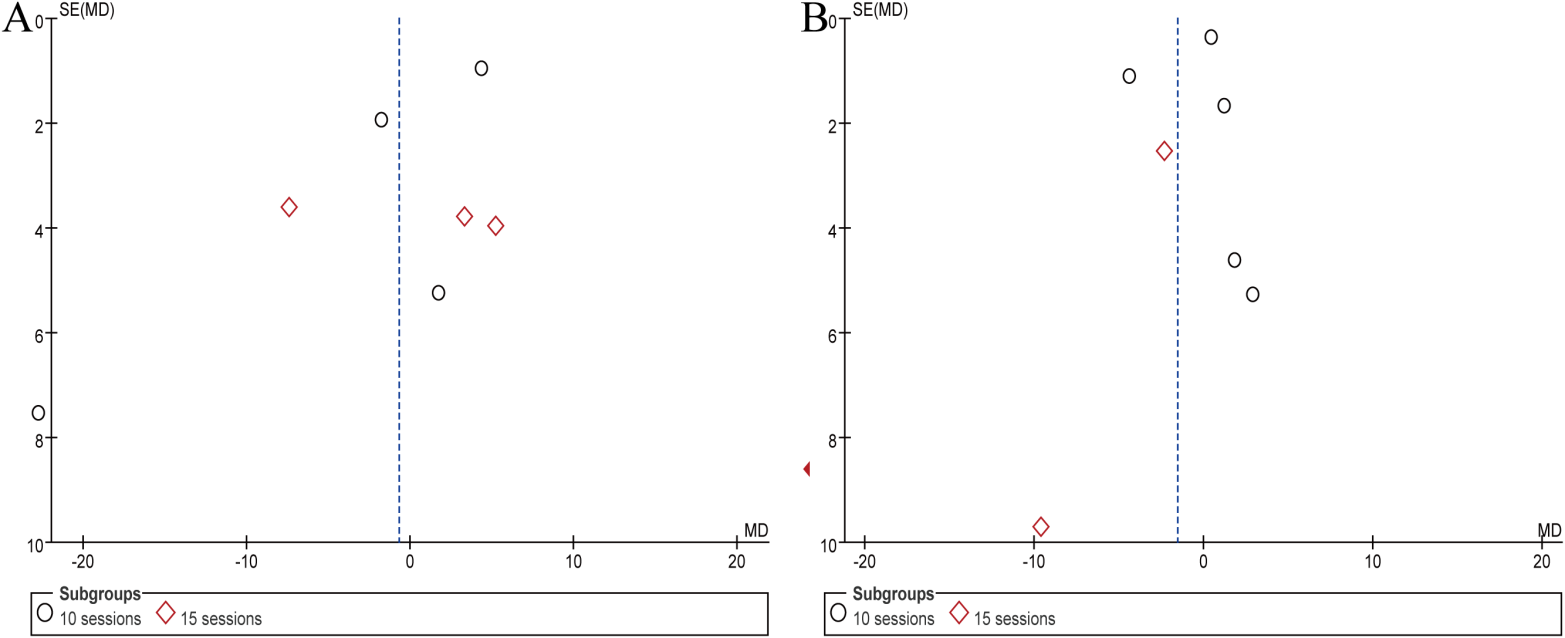
Results of publication bias assessment represented by PANSS and SANS. **(A)** PANSS, **(B)** SANS.

Considering the larger number of articles reporting on PANSS and SANS, the present study measured publication bias represented by PANSS and SANS. The funnel plot indicated that the literature did not distribute symmetrically on both sides of the dashed line (**Figure 6**). Frankly speaking, this study may be affected by publication bias.

### 3.7 Sensitivity analysis

We measured the sensitivity of each outcome using the leave one out method and found that the conclusions of this study are robust, meaning that the conclusions obtained after removing any single piece of literature are basically consistent with the original conclusions.

## 4 Discussion

### 4.1 Principal findings and clinical implications

The present study objectively evaluated the clinical efficacy of tDCS in the treatment of schizophrenia to the greatest extent possible through exploratory meta-analysis. The results showed that tDCS improved the PASAT score of schizophrenia patients, and intervention at 15 sessions or twice daily could also reduce the severity of the disease. Unfortunately, however, the therapeutic advantages of tDCS were not observed on PANSS, SANS, AHRS and CDSS. Therefore, frankly speaking, this is an exploratory research progress in the adjuvant use of tDCS in the treatment of schizophrenia. This study provided a new perspective and potential non-invasive intervention for the treatment of schizophrenia by verifying the effectiveness of TDCS in improving cognitive impairment and overall disease severity in patients with schizophrenia. It is worth noting that current research progress indicates that tDCS has the potential for home-based treatment, which means that future treatments will be more convenient, significantly reducing the burden on patients of frequent hospital visits, and improving the accessibility of treatment and patient compliance (41). Compared with drug therapy or traditional neuromodulation technology requiring special equipment and personnel, tDCS, as a technology with relatively simple operation, good tolerance and few side effects, is expected to be a useful supplement to the existing treatment schemes, and even provide new hope for some patients who have poor drug response or intolerance. What’s more, tDCS could complement existing antipsychotics—which show limited efficacy for cognitive symptoms—to bridge the current treatment gap. This study not only provided strong evidence support for the clinical application of tDCS in the treatment of schizophrenia, but also highlighted its unique advantages as a convenient and accessible treatment modality. By enhancing working memory, attention, and executive functions, tDCS may enable more effective engagement in psychosocial interventions, ultimately promoting long-term recovery. It laid the foundation for further in-depth research on the mechanism of action of tDCS, optimization of stimulation parameters, and exploration of its combined application with other therapies.

### 4.2 Comparison to previous studies

It is well known that tDCS has been controversial in improving cognitive function and reducing severity in patients with schizophrenia (42). In fact, in recent years, studies have confirmed that 10 sessions treatment can benefit patients with schizophrenia clinically (42). However, inconsistent with our findings, we found that receiving 15 sessions of tDCS reduced the severity of the disease, which may be the result of differences in the articles included. Previous meta-analyses have suggested that twice-daily tDCS can alleviate auditory hallucinations in schizophrenia (26), which is consistent with our research findings in frequency. Our conclusion is that twice-daily, not once-daily, tDCS can reduce the severity of schizophrenia. Previous studies have also found that increasing treatment frequency is associated with more significant efficacy (43).

### 4.3 Mechanisms by which tDCS improve cognitive function and reduce severity in patients with schizophrenia

We found that tDCS could significantly improve PASAT scores in patients with schizophrenia. PASAT is an effective neuropsychological assessment tool that can be used to evaluate cognitive function in patients with schizophrenia (44). Patients with schizophrenia commonly exhibit impairments across multiple cognitive domains, including attention, working memory, information processing speed, and executive function, and the PASAT is uniquely capable of simultaneously assessing these critical cognitive abilities. The DLPFC is a core region of the brain’s executive control network, primarily responsible for working memory, attention control, and cognitive flexibility (45). The cognitive components assessed by PASAT heavily rely on DLPFC function, as cognitive control requires the DLPFC to coordinate multiple processes, including auditory input, memory retrieval, arithmetic computation, and verbal output. This provides important insights into the neural mechanisms underlying tDCS-mediated improvements in cognitive function in patients with schizophrenia. Many scholars have explored the mechanism of tDCS from multiple perspectives.① Brain regions associated with cognitive function were selected for intervention: left dorsolateral prefrontal cortex (DLPFC), left temporo-parietal junction (TPJ), and orbitofrontal cortex. The left DLPFC is the target area to improve working memory (24), and it is also the target area most related to working memory. The above study found that for improved cognitive performance, it was necessary to place the anode electrode of tDCS on the left DLPFC, while the position of the cathode did not seem to matter much (24). Cognitive empathy is abnormal in patients with schizophrenia, and the left TPJ is the key area to regulate cognitive empathy. Patients with schizophrenia have reduced cortical thickness in the TPJ (46). Moreover, patients with schizophrenia showed an abnormal pattern of decreased activation in the TPJ when performing cognitive empathy tasks (47). First-episode schizophrenia patients show smaller orbitofrontal cortical volume (48), reduced cortical plasticity (49), and decreased thalamo-orbitofrontal cortical pathway connectivity (50), leading to altered neural signals. What’s more, tDCS can improve the motor skills learning by activating different brain regions (51) and attention function, particularly in improving executive effect performance (52, 53). ② Regulating neuronal activity and promoting neural plasticity. A recent study suggested that tDCS improves cognitive function in patients with schizophrenia by regulating internal neural activity and networks through externally applied currents, thereby regulating neural plasticity, particularly semantic processing deficits (54). In addition, there are studies suggesting that tDCS induces neurochemical changes through weak currents and regulates the excitability of neurons (55). Recently, it has been suggested that working memory deficits in schizophrenics are affected by irregularities in the posterior parietal cortex, in addition to being related to the DLPFC. What’s more, tDCS can improve the working memory level of schizophrenic patients by stimulating the DLPFC and the posterior parietal cortex to enhance the function of N-methyl-d-aspartate receptor (56). In fact, it has been demonstrated that tDCS enhances neuroplasticity and learning (6, 57).

### 4.4 Strength and limitation

Compared to previous meta-analyses, this study has significant advantages. Firstly, we strictly controlled the diagnostic criteria when screening literature. The previous meta-analysis focused on schizophrenia, but included patients with other diseases, such as schizoaffective disorder, delusional disorder, affective disorder and borderline personality disorder (26–29, 42, 58–61). We believe that such articles would add many confounding factors, making the aggregated data unreliable. Secondly, we referred to the previous literature (26), according to the sessions and frequency of subgroup analysis, so that the conclusion of the study is more detailed, and is of more guiding value for clinical operation. Moreover, recent studies have shown that tDCS has great potential in the field of neurocognition and can also have an impact on updating contingency based associations (62–64). Frankly, our research has some shortcomings. First, although we precisely controlled for schizophrenia in disease diagnosis, schizophrenia can be further subdivided into multiple subtypes, such as paranoid schizophrenia and disorganized schizophrenia. The proportion of different subtypes may also be one of the sources of heterogeneity in this study. Second, we found, when reading the literature, that the included studies accounted for handedness, and the proportion of left and right handedness may also be a factor of bias. Third, although we observed significant therapeutic effects on CGI and PASAT, tDCS did not demonstrate therapeutic advantages in other outcomes—a divergence that may stem from differences in the targeted domains of these measures, as well as the regionally specific and pathway-dependent mechanisms of tDCS. Due to the inconvenience of data merging, we did not quantitatively analyze the adverse event and discontinuation. Therefore, we speculate that tDCS may not be effective in every symptom group of schizophrenia, and the mechanism of tDCS needs further exploration. A fourth limitation of our study stems from the relatively small number of eligible documents identified, despite our stringent inclusion criteria aimed at enhancing research reliability. This rigorous screening process, combined with the inherently small sample sizes of some original studies, resulted in a relatively weak foundation for our pooled data. Consequently, the findings of this meta-analysis should be viewed as exploratory rather than providing definitive or conclusive evidence. Furthermore, the presence of high heterogeneity and significant publication bias represents crucial factors that diminish the strength of our conclusions. High heterogeneity indicates substantial variations among the included studies concerning design, intervention protocols, or participant characteristics, which mandates particular caution when interpreting pooled analysis results. Concurrently, the observed publication bias suggests that positive findings might have been preferentially published, potentially distorting the true effect size estimation. These elements further underscore the exploratory nature of this study, rather than offering definitive conclusions to guide clinical practice. Future investigations must address these challenges through more rigorously designed and comprehensively reported studies to provide more robust evidence regarding tDCS efficacy.

### 4.5 Inspiration for the future

Although there is an emerging awareness regarding the use of tDCS for the treatment of schizophrenia, and tDCS has begun to appear in clinical guidelines (65), the current exploration is still in its early stages. In addition to this study’s findings that tDCS has an effect on cognitive function, more comprehensive trial protocols are needed for other core symptoms, particularly in conjunction with other intervention methods.

## 5 Conclusion

While this exploratory meta-analysis provides initial insights, the available evidence is not yet conclusive regarding the definitive efficacy of tDCS in enhancing cognitive function among individuals with schizophrenia. Encouragingly, our preliminary exploratory analyses suggest that tDCS may possess therapeutic utility. To advance this field, there is a pressing need for future investigations to implement more standardized tDCS stimulation protocols and conduct larger, well-designed randomized controlled trials that can yield more definitive evidence for its clinical application, particularly with respect to other outcomes, such as PANSS, SANS, AHRS, and CDSS.

## Data Availability

The original contributions presented in the study are included in the article/**Supplementary Material**. Further inquiries can be directed to the corresponding author/s.

## References

[1] Sang N Fan Y Chen H Cui H Wei Y Tang X . Gender differences in cognitive performance among young adults with first-episode schizophrenia in China. Schizophr Res Cogn. (2025) 40:100353. doi: 10.1016/j.scog.2025.100353, PMID: 40028175 PMC11872115

[2] Wang X Wang R Bian C Liu F Tang M Zhang Y . Sleep quality, psychological resilience, family resilience, social support, and mental disability in patients with chronic schizophrenia: a cross-sectional study. Schizophr Res. (2024) 274:199–205. doi: 10.1016/j.schres.2024.09.020, PMID: 39341099

[3] Salahuddin NH Schutz A Pitschel-Walz G Mayer SF Chaimani A Siafis S . Psychological and psychosocial interventions for treatment-resistant schizophrenia: a systematic review and network meta-analysis. Lancet Psychiatry. (2024) 11:545–53. doi: 10.1016/S2215-0366(24)00136-6, PMID: 38879276

[4] Zu B Pan C Wang T Huo H Li W An L . Development and validation of a recurrence risk prediction model for elderly schizophrenia patients. BMC Psychiatry. (2025) 25:73. doi: 10.1186/s12888-025-06514-y, PMID: 39856611 PMC11762862

[5] Yao G Zeng J Huang Y Lu H Ping J Wan J . Discovery of biological markers for schizophrenia based on metabolomics: a systematic review. Front Psychiatry. (2025) 16:1540260. doi: 10.3389/fpsyt.2025.1540260, PMID: 40225847 PMC11985778

[6] Sehatpour P Kreither J Lopez-Calderon J Shastry AM De Baun HM Martinez A . Network-level mechanisms underlying effects of transcranial direct current stimulation (tDCS) on visuomotor learning in schizophrenia. Transl Psychiatry. (2023) 13:360. doi: 10.1038/s41398-023-02656-3, PMID: 37993420 PMC10665365

[7] Li J Lu X Du S Hu W Xiao D He B . Effects of varied rTMS frequencies on cognitive function in individuals with chronic schizophrenia: a double-blind randomized controlled trial. J Psychiatr Res. (2025) 189:33–41. doi: 10.1016/j.jpsychires.2025.05.052, PMID: 40479952

[8] Wei Y He S Luo P Su H Chen Y Qin S . Combining transcranial direct current stimulation with music therapy improves cognitive function in schizophrenia: study protocol for a randomized, double-blind, sham-controlled clinical trial. Front Psychiatry. (2025) 16:1543789. doi: 10.3389/fpsyt.2025.1543789, PMID: 40417274 PMC12100747

[9] Yuan X Li X Pang L Kang Y Hei G Zhang X . Association between purpureocillium, amino acid metabolism and cognitive function in drug-naive, first-episode schizophrenia. BMC Psychiatry. (2025) 25:524. doi: 10.1186/s12888-025-06965-3, PMID: 40405167 PMC12100923

[10] Debnath R Elyamany O Iffland JR Rauh J Siebert M Andraes E . Theta transcranial alternating current stimulation over the prefrontal cortex enhances theta power and working memory performance. Front Psychiatry. (2024) 15:1493675. doi: 10.3389/fpsyt.2024.1493675, PMID: 39876999 PMC11772280

[11] Wei X Shi Z Lan X Qin Z Mo Y Wu H . Transcranial alternating current stimulation for schizophrenia: a systematic review of randomized controlled studies. Front Psychiatry. (2023) 14:1308437. doi: 10.3389/fpsyt.2023.1308437, PMID: 38274423 PMC10808327

[12] Ma S Zhuang W Wang X Zhang D Wang H Han Q . Efficacy of transcranial direct current stimulation on cognitive function in patients with Parkinson’s disease: a systematic review and meta-analysis. Front Aging Neurosci. (2025) 17:1495492. doi: 10.3389/fnagi.2025.1495492, PMID: 40046783 PMC11880240

[13] Yun SJ Hyun SE Lee WH Oh B Seo HG . Comparison of stimulation sites enhancing dual-task performance using transcranial direct current stimulation in Parkinson’s disease. NPJ Parkinsons Dis. (2025) 11:19. doi: 10.1038/s41531-025-00869-5, PMID: 39827184 PMC11742881

[14] Yoon M Kim H Yoo YJ Im S Kim T Dhaher YY . In silico modeling of electric field modulation by transcranial direct current stimulation in stroke patients with skull burr holes: implications for safe clinical application. Comput Biol Med. (2025) 184:109366. doi: 10.1016/j.compbiomed.2024.109366, PMID: 39549527

[15] Lin X Li H Wu X Huang R . Effects of four non-invasive stimulations on swallowing function and quality of life of stroke patients—a network meta-analysis. Front Hum Neurosci. (2025) 19:1519660. doi: 10.3389/fnhum.2025.1519660, PMID: 40183069 PMC11966035

[16] Brunoni AR Padberg F . Spaced transcranial direct current stimulation for depression: the road less traveled. Am J Psychiatry. (2025) 182:231–3. doi: 10.1176/appi.ajp.20241088, PMID: 40022529

[17] Couture M Desbeaumes Jodoin V Bousseau E Sarshoghi A Nitsche MA Blumberger DM . Spaced transcranial direct current stimulation for major depression. Am J Psychiatry. (2025) 182:276–84. doi: 10.1176/appi.ajp.20240083, PMID: 39876681

[18] Wang J Yao X Ji Y Li H . Cognitive potency and safety of tDCS treatment for major depressive disorder: a systematic review and meta-analysis. Front Hum Neurosci. (2024) 18:1458295. doi: 10.3389/fnhum.2024.1458295, PMID: 39351069 PMC11439710

[19] Krauel K Brauer H Breitling-Ziegler C Freitag CM Luckhardt C Muhlherr A . Prefrontal transcranial direct current stimulation in pediatric attention-deficit/hyperactivity disorder: a randomized clinical trial. JAMA Netw Open. (2025) 8:e2460477. doi: 10.1001/jamanetworkopen.2024.60477, PMID: 39982727 PMC11846015

[20] Zhou Q Liu Z Yu C Wang Q Zhuang W Tang Y . Effect of combined treatment with transcranial direct current stimulation and repetitive transcranial magnetic stimulation compared to monotherapy for the treatment of chronic insomnia: a randomized, double-blind, parallel-group, controlled trial. BMC Med. (2024) 22:538. doi: 10.1186/s12916-024-03751-y, PMID: 39551773 PMC11571512

[21] Prathum T Chantanachai T Vimolratana O Laksanaphuk C Apiworajirawit I Aneksan B . A systematic review and meta-analysis of the impact of transcranial direct current stimulation on cognitive function in older adults with cognitive impairments: the influence of dosage parameters. Alzheimers Res Ther. (2025) 17:37. doi: 10.1186/s13195-025-01677-y, PMID: 39905569 PMC11796231

[22] Huang Y Zhang Y Zhang Y Mai X . Effects of transcranial direct current stimulation over the left primary motor cortex on verbal intelligence. Front Hum Neurosci. (2022) 16:888590. doi: 10.3389/fnhum.2022.888590, PMID: 35693542 PMC9177941

[23] Fan L Carrico S Zhu Y Ackerman RA Pinkham AE . Transcranial direct current stimulation improves paranoia and social functioning in schizophrenia: a randomized clinical trial. Biol Psychiatry. (2025) 1:1–9. doi: 10.1016/j.biopsych.2025.01.011, PMID: 39855408

[24] Garcia-Fernandez L Romero-Ferreiro V Padilla S Wynn R Perez-Galvez B Alvarez-Mon MA . Transcranial direct current stimulation (tDCS) enhances cognitive function in schizophrenia: a randomized double-blind sham-controlled trial. Psychiatry Res. (2025) 344:116308. doi: 10.1016/j.psychres.2024.116308, PMID: 39647260

[25] Lisoni J Baldacci G Nibbio G Zucchetti A Butti Lemmi Gigli E Savorelli A . Effects of bilateral, bipolar-nonbalanced, frontal transcranial direct current stimulation (tDCS) on negative symptoms and neurocognition in a sample of patients living with schizophrenia: results of a randomized double-blind sham-controlled trial. J Psychiatr Res. (2022) 155:430–42. doi: 10.1016/j.jpsychires.2022.09.011, PMID: 36182772

[26] Jiang W Cai D Sun C Yin F Goerigk S Brunoni AR . Adjunctive tDCS for treatment-refractory auditory hallucinations in schizophrenia: a meta-analysis of randomized, double-blinded, sham-controlled studies. Asian J Psychiatr. (2022) 73:103100. doi: 10.1016/j.ajp.2022.103100, PMID: 35430496

[27] Yu L Fang X Chen Y Wang Y Wang D Zhang C . Efficacy of transcranial direct current stimulation in ameliorating negative symptoms and cognitive impairments in schizophrenia: a systematic review and meta-analysis. Schizophr Res. (2020) 224:2–10. doi: 10.1016/j.schres.2020.10.006, PMID: 33129639

[28] Cheng PWC Louie LLC Wong YL Wong SMC Leung WY Nitsche MA . The effects of transcranial direct current stimulation (tDCS) on clinical symptoms in schizophrenia: a systematic review and meta-analysis. Asian J Psychiatr. (2020) 53:102392. doi: 10.1016/j.ajp.2020.102392, PMID: 32956993

[29] Sun C Jiang W Cai D Wang Z Sim K Ungvari GS . Adjunctive multi-session transcranial direct current stimulation for neurocognitive dysfunction in schizophrenia: a meta-analysis. Asian J Psychiatr. (2021) 66:102887. doi: 10.1016/j.ajp.2021.102887, PMID: 34740126

[30] Peng T Lin H Lee M Chen S . Statins as an adjuvant therapy for patients with schizophrenia: an up-to-date systematic review and meta-analysis. Gen Hosp Psychiatry. (2024) 89:75–83. doi: 10.1016/j.genhosppsych.2024.05.001, PMID: 38824832

[31] Jeon D Jung D Kim S Shim J Moon J Seo Y . Adjunct transcranial direct current stimulation improves cognitive function in patients with schizophrenia: a double-blind 12-week study. Schizophr Res. (2018) 197:378–85. doi: 10.1016/j.schres.2017.12.009, PMID: 30955702

[32] Dharani R Goyal N Mukherjee A Umesh S . Adjuvant high-definition transcranial direct current stimulation for negative symptoms in schizophrenia: a pilot study. J ECT. (2021) 37:195–201. doi: 10.1097/YCT.0000000000000756, PMID: 33661184

[33] Mondino M Jardri R Suaud-Chagny M Saoud M Poulet E Brunelin J . Effects of fronto-temporal transcranial direct current stimulation on auditory verbal hallucinations and resting-state functional connectivity of the left temporo-parietal junction in patients with schizophrenia. Schizophr Bull. (2016) 42:318–26. doi: 10.1093/schbul/sbv114, PMID: 26303936 PMC4753593

[34] Liu Y Li H Li W Wang Y Jiang J Cao X . Effects of transcranial direct current stimulation on brain changes and relation to cognition in patients with schizophrenia: a fMRI study. Brain Imaging Behav. (2022) 16:2061–71. doi: 10.1007/s11682-022-00676-z, PMID: 35781191

[35] Smith RC Md WL Wang Y Jiang J Wang J Szabo V . Effects of transcranial direct current stimulation on cognition and symptoms in chinese patients with schizophrenia(✰). Psychiatry Res. (2020) 284:112617. doi: 10.1016/j.psychres.2019.112617, PMID: 31806403

[36] Zhou Y Xia X Zhao X Yang R Wu Y Liu J . Efficacy and safety of transcranial direct current stimulation (tDCS) on cognitive function in chronic schizophrenia with tardive dyskinesia (TD): a randomized, double-blind, sham-controlled, clinical trial. BMC Psychiatry. (2023) 23:623. doi: 10.1186/s12888-023-05112-0, PMID: 37620825 PMC10464035

[37] Valiengo LDCL Goerigk S Gordon PC Padberg F Serpa MH Koebe S . Efficacy and safety of transcranial direct current stimulation for treating negative symptoms in schizophrenia: a randomized clinical trial. JAMA Psychiatry. (2020) 77:121–9. doi: 10.1001/jamapsychiatry.2019.3199, PMID: 31617873 PMC6802484

[38] Bose A Shivakumar V Agarwal SM Kalmady SV Shenoy S Sreeraj VS . Efficacy of fronto-temporal transcranial direct current stimulation for refractory auditory verbal hallucinations in schizophrenia: a randomized, double-blind, sham-controlled study. Schizophr Res. (2018) 195:475–80. doi: 10.1016/j.schres.2017.08.047, PMID: 28866447

[39] Palm U Keeser D Hasan A Kupka MJ Blautzik J Sarubin N . Prefrontal transcranial direct current stimulation for treatment of schizophrenia with predominant negative symptoms: a double-blind, sham-controlled proof-of-concept study. Schizophr Bull. (2016) 42:1253–61. doi: 10.1093/schbul/sbw041, PMID: 27098066 PMC4988747

[40] Lyu X Li Z Chen S Gu S Zhou Z Yang R . Transcranial direct current stimulation improves tardive dyskinesia in long-term hospitalized patients with chronic schizophrenia. Clin Neurophysiol. (2024) 166:20–30. doi: 10.1016/j.clinph.2024.07.006, PMID: 39084156

[41] Dragon K Abdelnaim MA Weber FC Heuschert M Englert L Langguth B . Treating depression at home with transcranial direct current stimulation: a feasibility study. Front Psychiatry. (2024) 15:1335243. doi: 10.3389/fpsyt.2024.1335243, PMID: 38501089 PMC10944921

[42] Adam O Blay M Brunoni AR Chang H Gomes JS Javitt DC . Efficacy of transcranial direct current stimulation to improve insight in patients with schizophrenia: a systematic review and meta-analysis of randomized controlled trials. Schizophr Bull. (2022) 48:1284–94. doi: 10.1093/schbul/sbac078, PMID: 35820035 PMC9673267

[43] Hyde J Carr H Kelley N Seneviratne R Reed C Parlatini V . Efficacy of neurostimulation across mental disorders: systematic review and meta-analysis of 208 randomized controlled trials. Mol Psychiatry. (2022) 27:2709–19. doi: 10.1038/s41380-022-01524-8, PMID: 35365806 PMC8973679

[44] Li J Xiao W Ye F Tang X Jia Q Zhang X . Brain-derived neurotrophic factor, sex hormones and cognitive decline in male patients with schizophrenia receiving continuous antipsychotic therapy. World J Psychiatry. (2023) 13:995–1004. doi: 10.5498/wjp.v13.i12.995, PMID: 38186728 PMC10768483

[45] Meng D Welton T Elsarraj A Morgan PS Das Nair R Constantinescu CS . Dorsolateral prefrontal circuit effective connectivity mediates the relationship between white matter structure and PASAT-3 performance in multiple sclerosis. Hum Brain Mapp. (2021) 42:495–509. doi: 10.1002/hbm.25239, PMID: 33073920 PMC7776003

[46] Bodnar M Hovington CL Buchy L Malla AK Joober R Lepage M . Cortical thinning in temporo-parietal junction (TPJ) in non-affective first-episode of psychosis patients with persistent negative symptoms. PLoS One. (2014) 9:e101372. doi: 10.1371/journal.pone.0101372, PMID: 24979583 PMC4076331

[47] Smith MJ Schroeder MP Abram SV Goldman MB Parrish TB Wang X . Alterations in brain activation during cognitive empathy are related to social functioning in schizophrenia. Schizophr Bull. (2015) 41:211–22. doi: 10.1093/schbul/sbu023, PMID: 24583906 PMC4266286

[48] Takayanagi Y Takahashi T Orikabe L Masuda N Mozue Y Nakamura K . Volume reduction and altered sulco-gyral pattern of the orbitofrontal cortex in first-episode schizophrenia. Schizophr Res. (2010) 121:55–65. doi: 10.1016/j.schres.2010.05.006, PMID: 20605415

[49] Jiao X Hu Q Tang Y Zhang T Zhang J Wang X . Abnormal global cortical responses in drug-naive patients with schizophrenia following orbitofrontal cortex stimulation: a concurrent transcranial magnetic stimulation-electroencephalography study. Biol Psychiatry. (2024) 96:342–51. doi: 10.1016/j.biopsych.2024.05.024, PMID: 38852897

[50] Kubota M Miyata J Sasamoto A Sugihara G Yoshida H Kawada R . Thalamocortical disconnection in the orbitofrontal region associated with cortical thinning in schizophrenia. JAMA Psychiatry. (2013) 70:12–21. doi: 10.1001/archgenpsychiatry.2012.1023, PMID: 22945538

[51] Qi S Liang Z Wei Z Liu Y Wang X . Effects of transcranial direct current stimulation on motor skills learning in healthy adults through the activation of different brain regions: a systematic review. Front Hum Neurosci. (2022) 16:1021375. doi: 10.3389/fnhum.2022.1021375, PMID: 36277051 PMC9582610

[52] Wei X Zhou R Zheng S Zhang Y Feng X Lu J . Network-based transcranial direct current stimulation enhances attention function in healthy young adults: a preliminary study. Front Hum Neurosci. (2024) 18:1421230. doi: 10.3389/fnhum.2024.1421230, PMID: 39175659 PMC11338793

[53] You G Pan X Li J Zhao S . Effects of transcranial direct current stimulation on modulating executive functions in healthy populations: a systematic review and meta-analysis. Front Hum Neurosci. (2024) 18:1485037. doi: 10.3389/fnhum.2024.1485037, PMID: 39734667 PMC11671507

[54] Pan W Li T Ma X Huo X . Effects of transcranial direct current stimulation combined with retrieval practice on semantic memory in patients with schizophrenia. BMC Psychiatry. (2025) 25:214. doi: 10.1186/s12888-025-06530-y, PMID: 40055696 PMC11889829

[55] Kronick J Sabesan P Burhan AM Palaniyappan L . Assessment of treatment resistance criteria in non-invasive brain stimulation studies of schizophrenia. Schizophr Res. (2022) 243:349–60. doi: 10.1016/j.schres.2021.06.009, PMID: 34183208

[56] Hou W Zhou F Wang Q Li H Qin X Ding Y . Effect of transcranial direct current stimulation with concurrent cognitive performance targeting posterior parietal cortex vs prefrontal cortex on working memory in schizophrenia: a randomized clinical trial. Transl Psychiatry. (2024) 14:279. doi: 10.1038/s41398-024-02994-w, PMID: 38977683 PMC11231223

[57] Auvichayapat N Auvichayapat P . Transcranial direct current stimulation in treatment of child neuropsychiatric disorders: ethical considerations. Front Hum Neurosci. (2022) 16:842013. doi: 10.3389/fnhum.2022.842013, PMID: 35874159 PMC9304992

[58] Kim J Iwata Y Plitman E Caravaggio F Chung JK Shah P . A meta-analysis of transcranial direct current stimulation for schizophrenia: “is more better? J Psychiatr Res. (2019) 110:117–26. doi: 10.1016/j.jpsychires.2018.12.009, PMID: 30639917

[59] Lee H Rast C Shenoy S Dean D Woodman GF Park S . A meta-analytic review of transcranial direct current stimulation (tDCS) on general psychopathology symptoms of schizophrenia; Immediate improvement followed by a return to baseline. Psychiatry Res. (2022) 310:114471. doi: 10.1016/j.psychres.2022.114471, PMID: 35227989 PMC8994865

[60] Narita Z Stickley A Devylder J Yokoi Y Inagawa T Yamada Y . Effect of multi-session prefrontal transcranial direct current stimulation on cognition in schizophrenia: a systematic review and meta-analysis. Schizophr Res. (2020) 216:367–73. doi: 10.1016/j.schres.2019.11.011, PMID: 31822431

[61] Lee EHM Chan PY Law EYL Lin JJX Hui CLM Chang WC . Efficacy of transcranial direct current stimulation (tDCS) as a treatment for persistent hallucinations in patients with schizophrenia: a systematic review and meta-analysis. Schizophr Res. (2018) 202:423–5. doi: 10.1016/j.schres.2018.06.069, PMID: 30150021

[62] van T Wout-Frank M Garnaat SL Faucher CR Arulpragasam AR Cole JE Philip NS . Transcranial direct current stimulation impairs updating of avoidance-based associative learning. Front Hum Neurosci. (2023) 17:1104614. doi: 10.3389/fnhum.2023.1104614, PMID: 37169017 PMC10164989

[63] Leon-Sarmiento FE Gonzalez-Castano A Rizzo-Sierra CV Aceros J Leon-Ariza DS Leon-Ariza JS . Neurophysics assessment of the muscle bioenergy generated by transcranial magnetic stimulation. Research (Wash D C). (2019) 2019:7109535. doi: 10.34133/2019/7109535, PMID: 31549082 PMC6750091

[64] Jin K Chen B Han S Dong J Cheng S Qin B . Repetitive transcranial magnetic stimulation (rTMS) improves cognitive impairment and intestinal microecological dysfunction induced by high-fat diet in rats. Research (Wash D C). (2024) 7:384. doi: 10.34133/research.0384, PMID: 38826566 PMC11140411

[65] Correll CU Schooler NR . Negative symptoms in schizophrenia: a review and clinical guide for recognition, assessment, and treatment. Neuropsychiatr Dis Treat. (2020) 16:519–34. doi: 10.2147/NDT.S225643, PMID: 32110026 PMC7041437

