# Supplementary material for: Adjunctive transcranial direct current stimulation for cognitive improvement in schizophrenia: insights from a systematic review and exploratory meta-analysis
Source: Front Psychiatry. 2025 Jul 11;16:1617068. doi: 10.3389/fpsyt.2025.1617068 (PMC12289641; doi:10.3389/fpsyt.2025.1617068)
Supplement: Supplementary file 1 [file DataSheet1.docx]

**Pubmed**

#1 ((((((((((((((((((((((((tDCS[MeSH Terms]) OR (Anodal Stimulation Transcranial Direct Current Stimulation[MeSH Terms])) OR (Anodal Stimulation tDCS[MeSH Terms])) OR (Anodal Stimulation tDCSs[MeSH Terms])) OR (Stimulation tDCS, Anodal[MeSH Terms])) OR (Stimulation tDCSs, Anodal[MeSH Terms])) OR (tDCS, Anodal Stimulation[MeSH Terms])) OR (tDCSs, Anodal Stimulation[MeSH Terms])) OR (Cathodal Stimulation Transcranial Direct Current Stimulation[MeSH Terms])) OR (Cathodal Stimulation tDCS[MeSH Terms])) OR (Cathodal Stimulation tDCSs[MeSH Terms])) OR (Stimulation tDCS, Cathodal[MeSH Terms])) OR (Stimulation tDCSs, Cathodal[MeSH Terms])) OR (tDCS, Cathodal Stimulation[MeSH Terms])) OR (tDCSs, Cathodal Stimulation[MeSH Terms])) OR (Transcranial Alternating Current Stimulation[MeSH Terms])) OR (Transcranial Random Noise Stimulation[MeSH Terms])) OR (Repetitive Transcranial Electrical Stimulation[MeSH Terms])) OR (Transcranial Electrical Stimulation[MeSH Terms])) OR (Electrical Stimulations, Transcranial[MeSH Terms])) OR (Electrical Stimulation, Transcranial[MeSH Terms])) OR (Stimulations, Transcranial Electrical[MeSH Terms])) OR (Stimulation, Transcranial Electrical[MeSH Terms])) OR (Transcranial Electrical Stimulations[MeSH Terms])) OR (Transcranial alternating current stimulation[MeSH Terms])

#2 (((((Schizophrenias[MeSH Terms]) OR (Dementia Praecox[MeSH Terms])) OR (Schizophrenic Disorders[MeSH Terms])) OR (Disorder, Schizophrenic[MeSH Terms])) OR (Disorders, Schizophrenic[MeSH Terms])) OR (Schizophrenic Disorder[MeSH Terms])

#3 #1 AND #2

**Embase**

#1 tdcs:ab,ti

#2 'anodal stimulation transcranial direct current stimulation':ab,ti

#3 'anodal stimulation tdcs':ab,ti

#4 'anodal stimulation tdcss':ab,ti

#5 'stimulation tdcs, anodal':ab,ti

#6 'stimulation tdcss, anodal':ab,ti

#7 'tdcs, anodal stimulation':ab,ti

#8 'tdcss, anodal stimulation':ab,ti

#9 'cathodal stimulation transcranial direct current stimulation':ab,ti

#10 'cathodal stimulation tdcs':ab,ti

#11 'cathodal stimulation tdcss':ab,ti

#12 'stimulation tdcs, cathodal':ab,ti

#13 'stimulation tdcss, cathodal':ab,ti

#14 'tdcs, cathodal stimulation':ab,ti

#15 'tdcss, cathodal stimulation':ab,ti

#16 'transcranial alternating current stimulation':ab,ti

#17 'transcranial random noise stimulation':ab,ti

#18 'repetitive transcranial electrical stimulation':ab,ti

#19 'transcranial electrical stimulation':ab,ti

#20 'electrical stimulations, transcranial':ab,ti

#21 'electrical stimulation, transcranial':ab,ti

#22 'stimulations, transcranial electrical':ab,ti

#23 'stimulation, transcranial electrical':ab,ti

#24 'transcranial electrical stimulations':ab,ti

#25 #1 OR #2 OR #3 OR #4 OR #5 OR #6 OR #7 OR #8 OR #9 OR #10 OR #11 OR #12 OR #13 OR #14 OR #15 OR #16 OR #17 OR #18 OR #19 OR #20 OR #21 OR #22 OR #23 OR #24

#26 Schizophrenias

#27 Dementia Praecox

#28 Schizophrenic Disorders

#29 Disorder, Schizophrenic

#30 Disorders, Schizophrenic

#31 Schizophrenic Disorder

#32 #26 OR #27 OR #28 OR #29 OR #30 OR #31

#33 #25 AND #32

**WOS**

#1 (((((((((((((((((((((((TS=(tDCS)) OR TS=(Anodal Stimulation Transcranial Direct Current Stimulation)) OR TS=(Anodal Stimulation tDCS)) OR TS=(Anodal Stimulation tDCSs)) OR TS=(Stimulation tDCS, Anodal)) OR TS=(Stimulation tDCSs, Anodal)) OR TS=(tDCS, Anodal Stimulation)) OR TS=(tDCSs, Anodal Stimulation)) OR TS=(Cathodal Stimulation Transcranial Direct Current Stimulation)) OR TS=(Cathodal Stimulation tDCS)) OR TS=(Cathodal Stimulation tDCSs)) OR TS=(Stimulation tDCS, Cathodal)) OR TS=(Stimulation tDCSs, Cathodal)) OR TS=(tDCS, Cathodal Stimulation)) OR TS=(tDCSs, Cathodal Stimulation)) OR TS=(Transcranial Alternating Current Stimulation)) OR TS=(Transcranial Random Noise Stimulation)) OR TS=(Repetitive Transcranial Electrical Stimulation)) OR TS=(Transcranial Electrical Stimulation)) OR TS=(Electrical Stimulations, Transcranial)) OR TS=(Electrical Stimulation, Transcranial)) OR TS=(Stimulations, Transcranial Electrical)) OR TS=(Stimulation, Transcranial Electrical)) OR TS=(Transcranial Electrical Stimulations)

#2 (((((TS=(Schizophrenias)) OR TS=(Dementia Praecox)) OR TS=(Schizophrenic Disorders)) OR TS=(Disorder, Schizophrenic)) OR TS=(Disorders, Schizophrenic)) OR TS=(Schizophrenic Disorder)

#3 #1 AND #2
