# Supplementary material for: Adjunctive transcranial direct current stimulation for cognitive improvement in schizophrenia: insights from a systematic review and exploratory meta-analysis
Source: Front Psychiatry. 2025 Jul 11;16:1617068. doi: 10.3389/fpsyt.2025.1617068 (PMC12289641; doi:10.3389/fpsyt.2025.1617068)
Supplement: Supplementary file 2 [file Table1.docx]

Supplementary Table 1 results of GRADE assessment for each outcome

| **Outcome** | **Certainty assessment** | | | | | | | **№ of patients** | | **Effect** | **Certainty** | **Importance** |
| --- | --- | --- | --- | --- | --- | --- | --- | --- | --- | --- | --- | --- |
|  | **№ of studies** | **Study design** | **Risk of bias** | **Inconsistency** | **Indirectness** | **Imprecision** | **Other considerations** | **active tDCS** | **sham tDCS** | **Absolute (95% CI)** |  |  |
| **PASAT** | 2 | randomised trials | not serious | not serious | not serious | serious^b^ | none | 40 | 32 | SMD: **1.72** (1.17 to 2.28) | ⨁⨁⨁◯Moderate^a^ | Critical |
| **PANSS** | 7 | randomised trials | not serious | serious^a^ | not serious | serious^b^ | publication bias strongly suspected,dose response gradient | 183 | 174 | SMD: **0.04** (0.45 to 0.53) | ⨁⨁◯◯Low^a,b^ | Important |
| **SANS** | 8 | randomised trials | not serious | seriousa | not serious | seriousb | publication bias strongly suspected,dose response gradient | 190 | 181 | SMD: 0.21 (0.59 to 0.17) | ⨁⨁◯◯Low^a,b^ | Important |
| **AHRS** | 3 | randomised trials | not serious | seriousa | not serious | seriousb | none | 73 | 75 | SMD: 0.52 (1.7 to 0.66) | ⨁⨁◯◯Low^a,b^ | Important |
| **CDSS** | 2 | randomised trials | not serious | not serious | not serious | not serious | none | 75 | 77 | SMD: 0.19 (0.51 to 0.13) | ⨁⨁⨁⨁High | Important |
| **CGI** | 3 | randomised trials | not serious | seriousa | not serious | seriousb | none | 57 | 59 | SMD: 0.53 (1.48 to 0.41) | ⨁⨁◯◯Low^a,b^ | Important |

Note: a. Significant heterogeneity, b. Small sample size. CI: confidence interval; SMD: standardised mean difference; PASAT: the Paced Auditory Serial Addition Task; PANSS: the Positive and Negative Syndrome Scale; SANS: the Scale for the Assessment of Negative Symptoms; AHRS: the Auditory Hallucinations Rating Scale;CDSS: Calgary Depression Scale for Schizophrenia; CGI: Clinical Global Impression-Schizophrenia scale.
